# Genomic landscapes of ovarian clear cell carcinoma from latin countries reveal aberrations linked to survival and progression

**DOI:** 10.1186/s12885-023-11095-8

**Published:** 2023-07-03

**Authors:** Mariana de Paiva Batista, Martín Roffé, Ignacio Romero, José Antonio López-Guerrero, Carmen Illueca, Raquel Lopez, Alexandre André Balieiro Anastácio da Costa, Louise De Brot, Juan Pablo Molina, Laura Barboza, Fernanda Maris Peria, Fernando Chaud, Ana Silvia Gouvêa Yamada, Andres Poveda, Eduardo Magalhães Rego

**Affiliations:** 1 Department of Internal Medicine, Medical School of Ribeirão Preto and Center for Cell Based Therapy, University of São Paulo, 14051-140, Tenente Catao Roxo, 2501, Ribeirão Preto, Brazil; 2 Children’s Hospital of Eastern Ontario Research Institute, Ottawa, ON Canada; 3 Gynecological Oncology Area, Valencian Institute of Oncology Foundation, Valencia, Spain; 4 Department of Gynecologic Oncology, A.C.Camargo Cancer Center, São Paulo, Brazil; 5 Department of Pathology, A.C.Camargo Cancer Center, São Paulo, Brazil; 6 Medical Oncology Service, México Hospital, CCSS, San José, Costa Rica; 7 Pathological Anatomy Service, San Juan de Dios Hospital, San José, Costa Rica; 8 Oncogynecologic Department, Quironsalud Hospital, Valencia, Spain

**Keywords:** Clear cell carcinoma of the Ovary, OncoScan, Latin Countries, Overall survival, Homologous recombination Deficiency

## Abstract

**Background:**

Ovarian clear cell carcinomas (OCCCs) are rare, aggressive and chemoresistant tumors. Geographical and ethnic differences in the incidence of OCCC have been reported with a higher incidence in Asiatic countries. There is a paucity of information regarding OCCC in Latin America (LA) and other countries.

**Methods:**

Here, we characterized two cohorts of 33 patients with OCCC from LA (24 from Brazil and 9 from Costa Rica) and a cohort of 27 patients from Spain. Genomic analysis was performed for 26 OCCC using the OncoScan platform. Tumors were classified according to their genomic landscapes into subgroups. Clinical parameters were related to the frequency of genomic aberrations.

**Results:**

The median overall survival (OS) was not significantly different between the cohorts. Genomic landscapes were characterized by different homologous recombination deficiency (HRD) levels. No difference in the distribution of genomic landscapes profiles was detected between patients from the different cohorts. OCCCs with *MYC*-amplified tumors harboring a concomitant loss of a region in chromosome 13q12-q13 that includes the *BRCA2* gene had the longest OS. In contrast, patients carrying a high number (> 30) of total copy number (CN) aberrations with no concomitant alterations in *MYC* and *BRCA2* genes presented the shortest OS. Furthermore, amplification of the *ASH1L* gene was also associated with a shorter OS. Initial-stage OCCCs with early progression were characterized by gains in the *JNK1* and *MKL1* genes.

**Conclusions:**

Our results provide new data from understudied OCCC populations and reveal new potential markers for OCCCs.

**Supplementary Information:**

The online version contains supplementary material available at 10.1186/s12885-023-11095-8.

## Introduction

Ovarian clear cell carcinoma (OCCC) accounts for 5–25% of all epithelial ovarian cancer (EOC) cases [1, 2]. Reported objective response rates to conventional platinum chemotherapy in OCCC are 11.1% compared to 72.5% in high-grade serous ovarian carcinoma (HGSC) [2, 3], representing an EOC of poorer prognosis, which is especially evident at advanced stages [1, 4]. Clinically, OCCCs are commonly associated with endometriosis [5], which is considered a direct precursor of clear cell carcinoma [6] and shows a higher incidence of thromboembolic events (TEEs) [6].

The overall frequency of OCCC in Asiatic populations (10.3–25%) [7–10] is higher than that in North America (12.2%) [11] and Europe (2–8%) [12, 13]. The reasons for this disparity remain unknown; however, some reports suggest genetic determinants [14]. The most frequent gene alterations in OCCC are in the *AT-rich interaction domain 1 A* (*ARID1A*) and *phosphatidylinositol-4,5-bisphosphate 3-kinase catalytic subunit α* (*PIK3CA*) genes [15]. Both alterations frequently coexist and occur as an early event in OCCC development [16, 17]. Other molecular markers are used to distinguish OCCC from other EOC histotypes and include the expression of hepatocyte nuclear factor 1 homeobox B (HNF1B) and the absence of Wilms tumor protein 1 (WT1), estrogen receptor (ER) and progesterone receptor (PR) [18]. Unlike HGSC, OCCCs usually express wild-type TP53 protein and have a much lower frequency of *BRCA1* and *BRCA2* mutations [19].

The scarce information about genome-wide patterns of aberrations in OCCCs was obtained from studies focusing on individual cohorts with different geographical and ethnic origins [20–24]. Remarkably, two of the most frequent copy number (CN) alterations found in OCCCs, i.e., amplification of chromosomes 8q and 20q13.2 (including the *ZNF217* oncogene), showed different prevalence in OCCCs with different geographical origins [21]. To date, genome-wide CN alteration profiles of OCCCs have not been obtained for Latin American countries.

In this study, we sought to provide a comprehensive description of the molecular characteristics of ovarian clear cell carcinomas (OCCCs) from Brazil, Costa Rica, and Spain, a European country with strong cultural ties to Latin America. By integrating clinical, molecular markers, and genomic aberration data, we aim to highlight the unique features of OCCCs from under-represented geographical regions. Overall, our findings may contribute to improving outcomes for patients with OCCC and inform personalized treatment strategies.

## Materials and methods

### Selection of patients

Sixty patients older than 18 years old with a histological diagnosis of OCCC between 2000 and 2015 were recruited from institutions in three different countries: 7 patients from Hospital das Clínicas de Ribeirão Preto (HCRP) - Brazil; 17 patients from A.C.Camargo Cancer Center (ACCCC) - Brazil; 9 patients from Hospital México (HM) – Costa Rica; and 27 patients from Instituto Valenciano de Oncología (IVO) – Spain. Since Brazil has been home to the largest Japanese population outside of Japan for over 100 years, particularly in the state of São Paulo where HCRP and ACCCC are located, three out of 24 patients from Brazil had Asian ancestry [25]. Fifty-seven samples were obtained from the primary tumor, and 4 corresponded to metastatic tissue after recurrence; matching primary and recurrence samples were available for one of the cases (Occ53). The samples were reviewed by two pathologists to confirm the diagnosis of pure OCCC histology.

### Clinical data

Tumor stage was defined according to the 2014 Ovarian Cancer FIGO staging [26]. Clinical data included date of diagnosis, progression, outcome and last follow-up, the occurrence of endometriosis and TEEs (**Supplementary File S1**). Overall survival (OS) and progression-free survival (PFS) were defined as the time interval from the date of diagnosis to the date of death or recurrence, respectively. Sensitivity to adjuvant treatment with platinum was established according to historical criteria supported by the statement of the Fourth Ovarian Cancer Consensus Conference (platinum sensitive: relapse ≥ 6 months after first-line platinum-based chemotherapy; platinum resistant: relapse < 6 months after first-line platinum-based chemotherapy) [27].

### Immunohistochemistry

Immunohistochemistry (IHC) was performed in tissue microarrays (TMAs) for the detection of ARID1A, HNF1B, PTEN, TP53, WT1, estrogen receptor (ER), progesterone receptor (PR) and mismatch repair proteins (MMR: MLH1, MSH2, MSH6, PMS2). Detailed information is provided in Supplementary Material and Methods.

### Microsatellite instability analysis

Microsatellite Instability (MSI) analysis was investigated using a PCR-based approach [28]. Detailed information is provided in Supplementary Material and Methods.

### Detection of *PIK3CA* gene mutations by real-time PCR

The cobas® PIK3CA Mutation Test (Roche Molecular Systems Inc.) and platform were used following the manufacturer’s instructions.

### OncoScan assay

DNA extracted from 26 primary tumors and one recurrent tumor was subjected to the SNP array OncoScan® FFPE (Thermo) following the manufacturer’s instructions. Detailed information is provided in Supplementary Material and Methods.

### OncoScan data processing

**Fig. 1:**
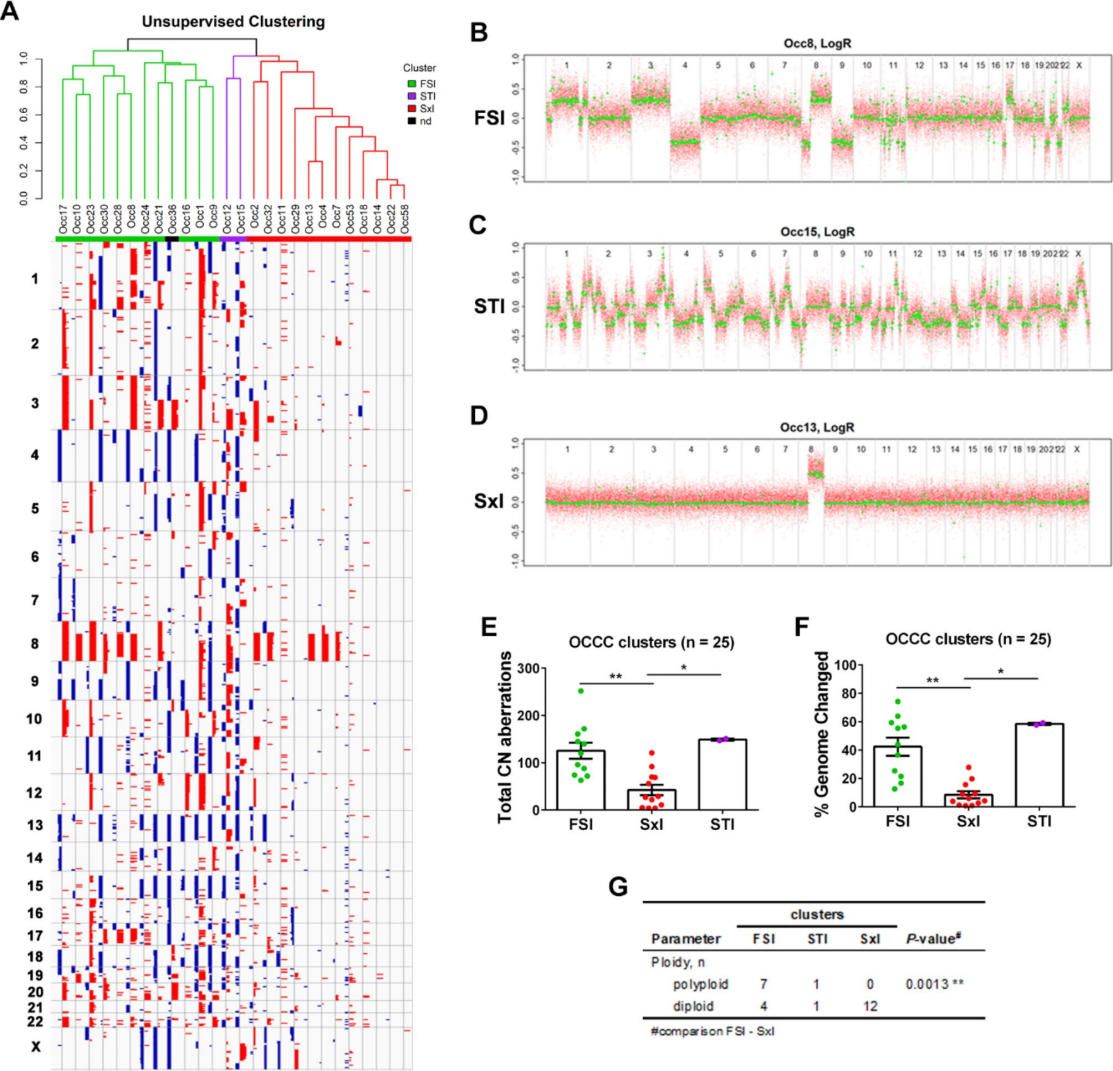
Genomic patterns for OCCC. **A** Unsupervised hierarchical clustering using segmented data from primary tumor samples of OCCC cases. The three clusters were named Firestorm-like (FSl), Sawtooth-like (STl) and Simplex-like (Sxl). **B-D** Log2 ratio profiles obtained with ASCAT for representative samples with (**B**) FSl, (**C**) STl or (**D**) Sxl patterns. **E-F** Graphs representing (**E**) total CN aberrations and (**F**) the percentage of genome changes for each cluster. Significant differences were analyzed by the Kruskal-Wallis test. **G** Ploidy was estimated from OncoScan analysis using the ASCAT algorithm. Polyploid samples were considered those with ploidy > 3 (n = 8), and diploid samples were considered those with ploidy 1.91–2.29 (n = 15). Fisher´s exact test was used to compare the FSl and Sxl clusters

CEL files containing the raw data were processed via the Affymetrix OncoScan Console (Thermo) (summarized in **Supplementary File S2**). The ASCAT algorithm was used to infer tumor ploidy, allele-specific copy numbers and segmentation [29]. The ASCAT package for R[30] used the following parameters: ascat.predictGermlineGenotypes(platform = “AffyOncoScan”) and ascat.runAscat(gamma = 0.9). Individual LRR are presented in Fig. 1, **Supplementary Fig.**S1 and S8. CN gains/amplifications were considered when the log R ratio (LRR) was > 0.1, and CN loss/deletions were considered when the LRR was < -0.1. The CNTools package for R and Fisher’s exact test were used to define differentially aberrant segments (*P*-value < 0.05). Integrative Genome Viewer (IGV) was used to inspect the genomic profiles [31].

### Estimation of the levels of homologous recombination deficiency

The estimation of the homology recombination deficiency (HRD)-associated genomic scars (loss-of-heterozygosity: HRD-LOH; large-scale transitions: HRD-LST; number of telomeric allelic imbalances: HRD-TAI; and a combined score: HRD-sum) was determined on the ASCAT output using the scarHRD package for R [32]. HRD-LST was defined as chromosomal breaks between adjacent regions of at least 10 Mb, with a distance between them not larger than 3 Mb [33]; HRD-TAI was defined as chromosomal breaks extending to the telomeric end of a chromosome [34]; and HRD-LOH was defined as regions of LOH exceeding 15 Mb that did not cover the whole chromosome [35].

### Statistical analyses

Univariate and multivariate Cox regression analyses were performed with the survival package for R. GraphPad Prism 9.0.1 software was used to apply the Kruskal–Wallis test followed by Dunn’s multiple comparisons. Contingency tables were analyzed by Fisher’s exact or χ2 tests, and *P*-values < 0.05 were considered statistically significant. Due to the reduced sample size, we were not able to apply a multiple comparison test without compromising the statistical power.

## Results

### Clinical characteristics of the OCCC cohorts

Patients of three different countries (Brazil, Costa Rica and Spain) were included in this study. There was no significant different in OS or PSF between the cohorts of the different countries (**Supplementary Fig.**S2). Overall, more than 60% of the patients in our cohorts were diagnosed at an initial FIGO stage (I and II). While 88% of the patients with endometriosis were diagnosed at the initial stage, only 57% of the patients without endometriosis were at an initial stage (*P*-value = 0.0342; Fisher’s exact test). At least 60% of the patients who received platinum-based chemotherapy displayed sensitivity to the treatment (Table 1). Univariate analysis of OS indicated that advanced (III or IV) Figo stage, platinum resistance or TEEs increased the risk for a deadly outcome (**Supplementary Table**S2). In a multivariate analysis, resistance to platinum treatment was the main variable associated with increased risk, followed by TEEs (**Supplementary Fig.**S3A). Regarding PFS, advanced-stage disease was associated with an increased risk for recurrence, but it was not an independent prognostic factor. The occurrence of TEEs showed a marginal association with an increased risk for progression (**Supplementary Table**S2 and Fig. S3B).

**Table 1:** Clinicopathological features

		Cohort		
Parameter	All patients	Brazil	Costa Rica	Spain
Number of cases	60	24	9	27
Age at diagnosis, y (range)	50 (29–75)	53 (29–75)	48 (36–70)	48 (31–74)
Asian ancestry, n (%)	3 (5)	3 (13)	0 (0)	0 (0)
FIGO stage, n (%)				
Initial (I/II)	39 (65)	15 (63)	6 (67)	18 (67)
III	17 (28)	8 (33)	2 (22)	7 (26)
IV	4 (7)	1 (4)	1 (11)	2 (7)
Endometriosis, n (%)	16 (27)	7 (29)	1 (11)	8 (30)
TEEs, n (%)	14 (23)	8 (33)	1 (11)	5 (19)
Sensitivity to platinum, n (%)				
sensitive	35 (66)	12 (60)	6 (75)	17 (68)
resistant	18 (34)	8 (40)	2 (25)	8 (32)
unknown	7	4	1	2
Progression, n (%)	36 (60)	15 (63)	3 (33)	18 (67)
Outcome, n (%)				
Alive	32 (53)	11 (46)	6 (67)	15 (56)
Dead	28 (47)	13 (54)	3 (33)	12 (44)
Median follow-up, m	36.3	39.7	15.6	36.3
(range)	(0.43–210.6)	(0.43–123.7)	(3.1–41.0)	(1.1-210.6)

*FIGO* International Federation of Gynecology and Obstetrics, *TEE* thromboembolic event

### Molecular characterization of the OCCC cohorts

HNF1B expression was observed in 90% of the patients with no significant differences between the cohorts (Table 2). ARID1A and WT1 showed no protein expression in approximately 29% and 91% of the patients, respectively. Furthermore, ER and PR protein expression was observed in 7% and 2% of the tumors, respectively. Incidence of abnormal TP53 protein expression (20%) in the entire cohort is compatible with the characteristic lower incidence of this alteration in OCCC when compared to HGSC [36]. The results obtained with the aforementioned molecular markers and morphological characteristics were as expected for the histopathological diagnosis of OCCC.

*PIK3CA* gene mutations were detected in 29% of our patients with predominant mutations in residue p.H1047, followed by mutation p.E543K. *PI3KCA* mutations were less frequent in cases detected in advanced stages III and IV (**Supplementary Table**S3). Interestingly, 61.5% of patients harboring *PIK3CA* mutations showed a concomitant loss of ARID1A expression, while negative ARID1A expression was observed only in 18.5% of patients with wild-type *PIK3CA* tumors (**Supplementary Table**S3). MSI was detected by PCR in 10 of 44 of the cases (22.7%), 8 of which were classified as MSI-high (**Supplementary File S1**). However, we did not detect a concomitant deficiency of MMR proteins by IHC (**Supplementary File S1**).

**Table 2:** Molecular markers in OCCC primary tumor samples

			Cohort		
Molecular marker	Method	All patients	Brazil	Costa Rica	Spain
HNF1B expression, n (%)	IHC				
positive		43 (90)	16 (80)	7 (100)	20 (95)
negative		5 (10)	4 (20)	0 (0)	1 (5)
unknown		9	1	2	6
WT1 expression, n (%)	IHC				
positive		5 (9)	3 (14)	1 (12)	1 (4)
negative		51 (91)	18 (86)	7 (88)	26 (96)
unknown		1	0	1	0
ER expression, n (%)	IHC				
positive		4 (7)	2 (10)	0 (0)	2 (7)
negative		53 (93)	19 (90)	9 (100)	25 (93)
PR expression, n (%)	IHC				
positive		1 (2)	0 (0)	0 (0)	1 (4)
negative		56 (98)	21 (100)	9 (100)	26 (96)
TP53 expression, n (%)	IHC				
aberrant		10 (20)	3 (17)	3 (37)	4 (15)
normal		42 (80)	14 (82)	5 (63)	23 (85)
unknown		5	4	1	0
ARID1A expression, n (%)	IHC				
positive		35 (71)	14 (74)	7 (87)	14 (64)
negative		14 (29)	5 (26)	1 (13)	8 (36)
unknown		8	2	1	5
*PIK3CA* mutation, n (%)	real-time PCR				
mutant		14 (30)	4 (25)	3 (37)	7 (32)
wild-type		32 (70)	12 (75)	5 (63)	15 (68)
unknown		11	5	1	5
*IHC* Immunohistochemistry					

### Whole-genome copy number analysis of OCCC

The OncoScan platform showed that the most frequent alteration (> 50% of the 26 samples) corresponded to the arm-level amplification of chromosome 8q, which includes the oncogene *MYC* (**Supplementary Fig.**S4A and **Files S3** and **S4**), followed by the focally amplified region 20q13.2, which harbors the putative oncogene *ZNF217* (**Supplementary Fig.**S4A). Recurrent gains (41% of the cases) were observed in 3q1.2-q13.12 and 3q26.2, with the latter containing several genes for kinases, such as *PIK3CA* [37]. Amplification of 17q12, containing *HNF1B* and 1q22, was observed in 38% of the patients, however, both focal- and broad-level amplification events were noticed. Loss of the 13q arm occurred in 44% of the OCCC patients (**Supplementary Fig.**S4A and **File S4**). The GISTIC algorithm defined significantly amplified peaks in *HNF1B* in cytoband 17q12 and *MECOM* in 3q26.2. A significantly deleted peak at cytoband 1p36.11 contains *ARID1A* (**Supplementary Fig.**S4B and **File S5**).

### Genomic alteration patterns in OCCC

OCCC samples showed remarkable heterogeneity of genomic alterations, ranging from cases with very few CN aberrations to samples with genomic changes in more than half of the genome (**Supplementary File S3**). Unsupervised hierarchical clustering using the LRRs of the segments as input was performed (Fig. 1A), and each cluster resembled the genomic patterns first described in breast cancer [38] and later defined for OCCCs. Tan et al. defined three genomic patterns: Firestorm (FS), Sawtooth (ST) and Simplex (Sx) [24]. The FS pattern is characterized by broad segments of duplication and deletion, usually comprising entire chromosomes or chromosome arms, with occasional isolated narrow peaks of amplifications. The ST pattern displays segments of duplication and deletion, often alternating and affecting all chromosomes characterizing a more complex pattern. The Sx pattern was characterized by occasional isolated narrow peaks of gains and deletions and essentially diploid genomes. In accordance, we named the clusters FS-like (FSl), ST-like (STl) and Sx-like (Sxl) (Fig. 1A-D). Twelve samples were classified as Sxl as they represented a reduced number of CN aberrations and a low percentage of the genome with changes (Fig. 1E **and F**). Only two samples (7.7%) clustered in the STl cluster, and they were the only samples showing deletions in chromosome 10q23.2-q25.2, which includes the tumor suppressor gene *PTEN*. One of the samples (Occ36) clustered in STl or FSl depending on the parameters used; due to this ambiguity, it was not classified (Fig. 1A**)**. Eleven samples were classified as FSl, and, remarkably, 9 of them showed amplification of the *ZNF217* gene (**Supplementary Fig.**S5A-C). Moreover, of the 8 polyploid samples in our analysis, 7 were FSl (Fig. 1G **and Supplementary File S3**). Even with such different characteristics of the clusters, no difference in OS or PFS was observed (**Supplementary Fig.**S5D and E).

### HRD is related to genomic patterns in OCCC

**Fig. 2:**
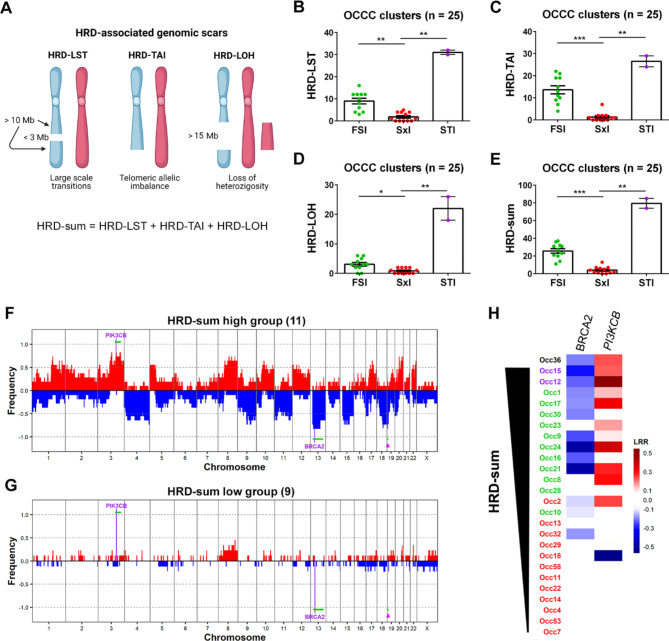
HRD in OCCC samples. **A** Data obtained with OncoScan were used to analyze genomic scars associated with HRD by the scarHRD package. The scheme shows the HRD signatures included in the analysis. HRD-LST: number of chromosomal breaks between adjacent regions of at least 10 Mb, with a distance between them not larger than 3 Mb; HRD-TAI: number of allelic imbalances that extend to the telomeric end of a chromosome; HRD-LOH: number of LOH regions exceeding 15 Mb that do not cover the whole chromosome; HRD-sum: combined score for HRD. **B-E** HRD-associated scores for the clusters of OCCC. Significant differences were analyzed by the Kruskal-Wallis test. **F-G** Samples were separated according to HRD-sum scores, and frequency plots were generated for the (**F**) HRD-sum high and (**G**) HRD-sum low groups of OCCC. Significantly altered genes and regions (green line) are indicated. The arrowhead points to a recurrent loss within chromosome 19p13.3 that occurs in HRD-sum high samples. **H** Summary of CN alterations in two genes recurrently altered in OCCCs with higher HRD-associated scores. HRD scores for sample Occ36 could not be obtained; however, from its resemblance with FSl/STl samples, it is expected that Occ36 is deficient for HR

To gain insight into the mechanisms underlying the distinct genomic patterns observed in OCCC, three HRD-associated genomic scars were estimated (Fig. 2A): HRD-LST [33]; HRD-TAI [34]; and HRD-LOH [35]. A combined score (HRD-sum) including the three signatures was then obtained [39]. As expected, genomic scars were practically absent in the Sxl cluster (Fig. 2B-D). The FSl cluster showed higher levels of HRD-TAI and HRD-LST and, to a lesser extent, HRD-LOH than the Sxl cluster. The two STl samples showed higher levels of all genomic scars, when compared to the FSl cluster, ranging from almost a 2-fold increase for HRD-TAI to 7-fold in HRD-LOH; however, due to the reduced number of samples, this difference was not statistically significant. The combined score, HRD-sum, perfectly matched the clustering based on genomic patterns, strongly pointing to HRD as one of the mechanisms involved (Fig. 2E). To investigate the genomic determinants associated with HRD, we divided the samples into two groups based on their HRD-sum levels: the HRD-sum high group, composed of the top eleven samples (HRD-sum levels ≥ 20, except for Occ36); and the HRD-sum low group, composed of the nine samples with the lowest HRD-sum levels (≤ 6; **Supplementary File S3 and Supplementary Fig.**S6A). Nine of 11 samples (82%) of the group with higher HRD-sum levels showed gains in the *PIK3CB* gene (cytoband 3q22.3) and in the recurrently amplified region 3q26.2 (Fig. 2F-G). Additionally, 82% of HRD-sum high samples showed losses of 13q12.12-q21.33, which includes the *BRCA2* gene, and 19p13.3. No difference in OS or PFS was identified for the HRD-sum high and HRD-sum low groups (**Supplementary Fig.**S6B and C). Remarkably, 8 of the 13 samples with the highest HRD-sum from the whole cohort showed concomitant loss of *BRCA2* and gain of *PIK3CB* (*P*-value = 0.0016 for Fisher’s exact test) (Fig. 2H).

### Genomic alterations associated with different OS outcomes

**Fig. 3:**
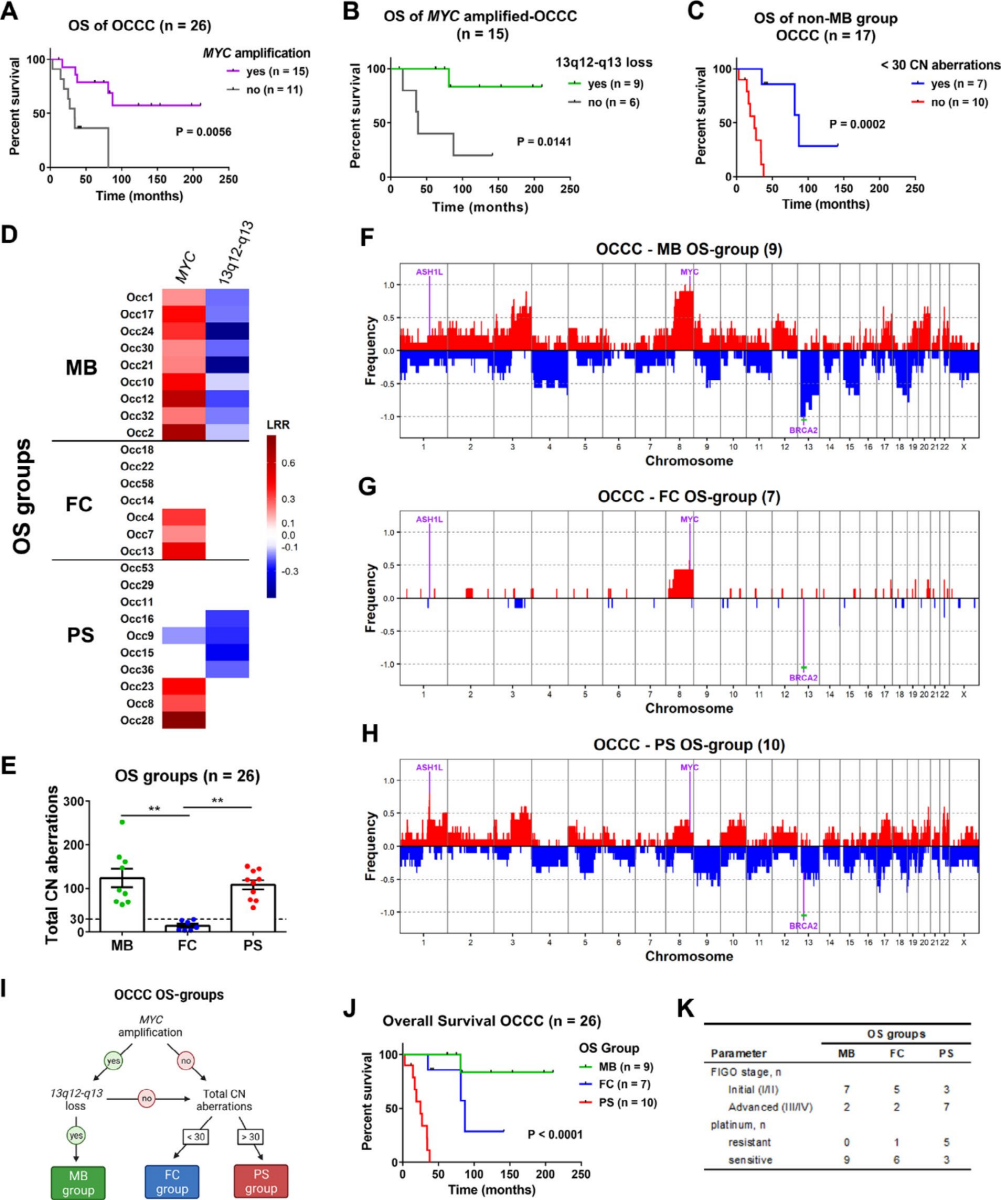
Genomic determinants associated with OS outcome in OCCC. Survival data were analyzed in relation to genomic aberrations detected by OncoScan. **A** Survival analysis showing that amplification of the oncogene *MYC* is associated with better survival. **B** Survival analysis showing a subgroup within *MYC*-amplified OCCCs with better prognosis that was defined by the concomitant loss of chromosome region 13q12-q13, which includes the *BRCA2* gene (**MB group**). **C** Survival analysis for OCCCs that do not bear concomitant *MYC* amplification and 13q12-q13 loss (non-MB OCCCs). A subgroup of OCCCs with few CN aberrations shows better survival (**FC group**). The OCCCs not belonging to either the MB or FC groups that showed the worst survival were named the **PS group. D** Summary of CN alterations in *MYC* and 13q12-q13. (**E**) Graph showing the total CN aberrations of each OS group. The dashed line was set at 30 CN aberrations. Significant differences were analyzed by the Kruskal-Wallis test. **F-H** Frequency plots were generated for the (**F**) MB, (**G**) FC and (**H**) PS OS groups. Significantly altered genes and regions (green line) are indicated. **I** Flowchart for the definition of OS groups (created with BioRender.com). **J** Survival analysis for the OS groups. **K** Summary of the Figo stage and platinum sensitivity of the samples within each OS group

Neither genomic patterns nor HRD levels were associated with OS outcome in OCCC. In this manner, we carried out comparisons between long- and short- survivors. The frequency of CN alterations in those two groups indicated that gain/amplification of the *MYC* gene was associated with better survival (Fig. 3A). Survival was even better in a subgroup of *MYC*-amplified tumors harboring a concomitant loss of a region in chromosome 13q12-q13, which includes the *BRCA2* gene; thus, this OS group of long-term survivors was named MB (“*MYC*-*BRCA2*”; Fig. 3B, D **and F**). A further comparison of OS was performed in the non-MB OCCCs, and better survival was observed in a subgroup carrying few (< 30) total CN aberrations (“few CN alterations” group; FC; Fig. 3C, E **and G**). The genomic pattern of all FC samples was Sxl, accordingly with lower percentages of genome changed and LOH (**Supplementary Fig.**S7A and B). The remaining samples showed the poorest survival (“poor survival” group; PS). Strikingly, amplification of the *ASH1L* gene was found in 8 (80%) of 10 PS samples, while *ASH1L* was amplified in only 3 (19%) of 16 non-PS samples (Fig. 3C **and H** and **Supplementary File S6**). The median OS of amplified *ASH1L* samples was 34.3 months versus 87.3 months for the remaining samples (*P*-value = 0.0407 and 0.0884 for Gehan-Breslow-Wilcoxon and log-rank tests, respectively; **Supplementary Fig.**S7C). Using the mentioned approach, the samples were classified into three OS groups (Fig. 3I) with different median OS times: 25 months for PS; 87.3 months for FC; and for the MB group, there was only one death event (Fig. 3J). All patients in the MB group were sensitive to platinum, and most of the patients in the PS group were diagnosed at stage III or IV (Fig. 3K). However, it is worth noting that patients with PS-OCCCs diagnosed at initial stages showed poor survival (**Fig.**S7D) and that patients with non-PS-OCCCs diagnosed at stage III showed a better prognosis (**Fig.**S7E).

### Genomic alterations associated with early progression of initial-stage OCCC

**Fig. 4:**
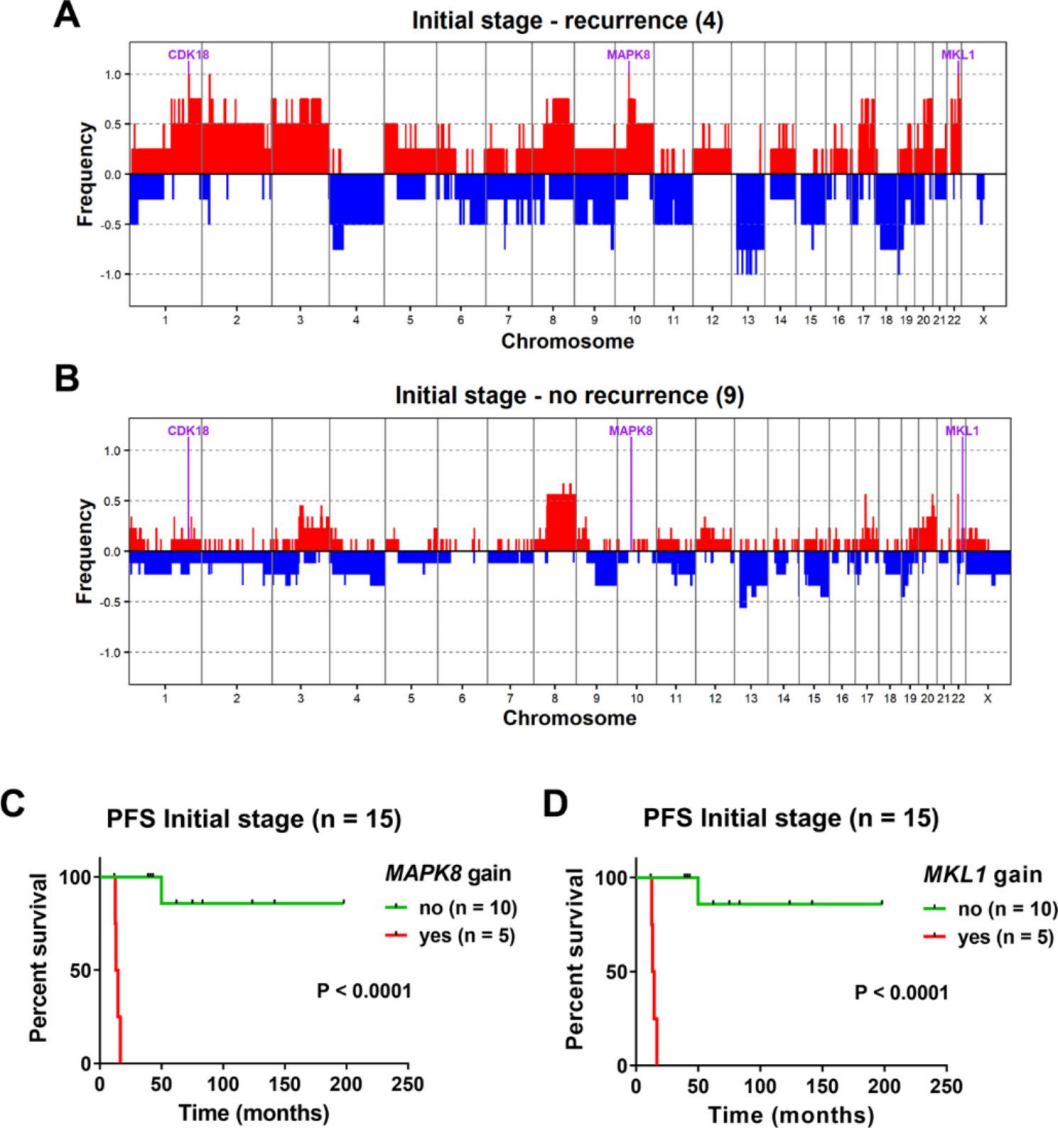
Genomic alterations associated with the progression of initial-stage OCCC. Frequency of CN alterations in (**A**) cases that recurred before 17 months and (**B**) cases that did not show recurrence in at least 39 months. Recurrently amplified genes in the cases showing recurrence are indicated. **C-D** PFS curve for all the initial-stage cases in our cohort after classification on the basis of (**C**) *MAPK8* or (**D**) *MKL1* amplification status

To identify the association between genetic alterations and PFS, a comparison between four initial-stage samples that recurred before 17 months and 8 samples that did not progress in at least 39 months was performed. We found amplifications in the *MAPK8* (cytoband 10q11.22), *MKL1* and *MCHR1* genes (cytoband 22q13.1-q13.2) in the group that recurred (Fig. 4A **and B, and Supplementary File S7**). A survival analysis on the basis of amplified *MAPK8* (Fig. 4C) or *MKL1* (Fig. 4D) status showed that one sample with delayed recurrence at 46.6 months did not show amplification of either gene. Interestingly, Occ53 showed MAPK8 amplification on recurrence (**Supplementary File S3**).

## Discussion

In the present study, the median age of diagnosis was 50 years, consistent with the earlier diagnoses of OCCCs compared to HGSC in the United States (median 55 vs. 64 years) [3]. Moreover, OCCC was detected earlier in women with endometriosis (median = 38 years, range 31–59), which is probably connected with the fact that most of those patients were diagnosed at the initial stage [40, 41]. To date, the most important prognostic factor for overall survival is the sensitivity to platinum-based chemotherapy, regardless of the stage of the tumor [2]. However, for predicting progression-free survival (PFS), an advanced stage of cancer has been identified as the main prognostic factor, with the occurrence of thromboembolic events (TEEs) also contributing to the prognosis. In our cohort, we found that endometriosis did not significantly impact either OS or PFS. This is in contrast to a previous report, which suggested that endometriosis could be a prognostic factor [42].

*PIK3CA* mutations are common in OCCC; accordingly, they were detected in 30% of our samples with a relatively lower frequency than reported in other populations [37, 43–50]. Our cohort also showed the concomitant loss of ARID1A expression associated with *PI3KCA* mutation, as previously observed [16]; those alterations are thought to occur at an early stage in the development of OCCC due to its presence in endometriosis precursor lesions [6]. In fact, we observed that endometriosis was more frequent in the presence of *PIK3CA* mutations and that both were associated with diagnosis at the initial stage. The cohort displayed an incidence of abnormal TP53 which is consistent with previously reported incidences in other populations [51, 52].

The genome-wide analysis of alterations by OncoScan was never used to detect CN alterations for this particular ovarian tumor type; the first studies of OCCCs were performed with aCGH, and currently, whole-exome sequencing is being performed. Despite the different reported techniques, the most common CN alterations were easily detected in our samples, including chromosome 8q and 20q13.2 amplification. The latter contains the *ZNF217* oncogene that was not associated with shorter PFS or OS (log-rank *P*-value = 0.3125 and 0.5571, respectively), as suggested before [53]. The OCCC samples were clearly separated into clusters depending on their genomic architecture (FSl, STl and Sxl) rather than on individual CN alterations. The finding that most of the polyploid samples were inside the FSl cluster and none were classified as Sxl suggests that the genomic instability associated with the FSl pattern can be related to the loss of the diploid state [54]. Interestingly, ploidy alterations are associated with outcome in ovarian cancer, including OCCC [55].

Amplification of *MYC* was identified in 57.7% of the samples, which is consistent with previous reports (40–64%) [20, 44, 46] and was associated with better survival. However, a concomitant loss of the chromosome 13 region containing *BRCA2*, in the MB group, was associated with outstanding good OS prognosis, suggesting that a synergistic interaction might exist. In fact, loss of *BRCA2* was a recurrent alteration in the samples within our cohort with high levels of HRD. A recent article by Wang et al. proposed a classification of ovarian tumors based on genomic signatures of aberrant DNA repair mechanisms rather than on histology [49]. In Wang et al., the subgroup H-HRD (high-HRD), characterized by enrichment of HRD signatures, showed better survival even in ovarian tumors without *BRCA1/BRCA2* mutations. Importantly, the H-HRD subgroup showed amplification of *MYC* and *MECOM* genes (3q26.2) [49], the latter being within a region we identified as recurrently amplified in the HRD-sum high group of OCCC patients. In this manner, the MB OS group of OCCCs we defined is reminiscent of the H-HRD; however, in the combined study of Wang et al., most H-HRD samples were HSGC, and no OCCC was present [49]. Our hypothesis for this observation is that when the different histotypes are studied together, the greater levels of alterations found in HGSC mask similar alterations at lower levels in the other histotypes. This is clear from the differences observed in genomic scar levels between the FSl and STl clusters, where the latter showed exaggerated levels of all HRD signatures, in particular of HRD-LOH, relative to the FSl cluster. A recent study by Pesenti et al. classified a cohort of Italian-origin stage I epithelial ovarian cancer, including OCCCs, based on their genomic instability patterns, regardless of histological subtype [56]. The three genomic instability patterns defined by Pesenti et al. - “stable”, “unstable”, and “highly unstable” - are analogous to the Sx, FS, and ST genomic patterns, respectively, previously defined by Tan et al. [24]. OCCCs with lower levels of genomic alterations in our cohort were more prevalent than in the study by Pesenti et al. However, differences in sample classification between the two studies may have contributed to this discrepancy.

Synthetic lethality induced by PARP inhibitors in tumor cells with HRD is supposed to be a groundbreaking therapeutic strategy [57] in particular, for HGSC where ~ 50% of the cases have HRD [36]. Recently, mutations in 16 HR-associated genes were tested in a Japanese cohort, and 28% of the OCCCs showed alterations, which suggests that more patients could be selected for treatment with PARP inhibitors [58]. We observed that loss of *BRCA2* and gain of *PIK3CB* genes were present in OCCCs with higher HRD-sum levels, which includes most of the cases in the FSl and STI clusters. In this manner, it would be interesting to verify what level of HRD-sum is necessary for the use of PARP inhibitors in OCCCs and to verify whether CN loss of *BRCA2* is associated with that. Furthermore, the observed CN gain of the *PIK3CB* gene in HRD-sum high OCCCs might be linked to the promising results observed with the combination of inhibitors for the PI3K pathway and PARP [59, 60]. It has been reported that ovarian cancer patients with mutations in HR-associated genes have higher platinum sensitivity and prolonged overall survival [61]. In our cohorts, HRD-sum and CN loss of *BRCA2* were not necessarily associated with longer OS or platinum sensitivity. In fact, all the patients with concomitant gain in *MYC* and loss of *BRCA2* were sensitive to platinum-based treatment, and all of them showed a better prognosis.

The majority of the cases of the poorest OS group (PS group) showed amplification of the *ASH1L* gene on chromosome 1q22. This gene codes for a histone lysine methyltransferase that can mono- or di-methylate histone H3 lysine 36 (H3K36) [62] and is part of the Trithorax group of chromatin proteins that act as epigenetic regulators [63]. Recently, ASH1L function has been linked to leukemogenesis in mixed-lineage leukemia [57] and acute myelogenous leukemia [64]. Furthermore, *ASH1L* gene was identified as a driver gene liver cancer [65]. Also, congruent *ASH1L* gene amplification and mRNA up-regulation was reported in hepatocellular carcinoma [66]. Additionally, ASH1L is overexpressed in anaplastic thyroid cancer (ATC), contributing to its aggressiveness [67]. As in other tumor types, ASH1L might be associated with OCCC biology, and since epigenetic regulators are considered important targets for cancer treatment, our observation opens a new research opportunity to define the role of ASH1L in OCCC [68].

An important challenge in OCCC is to predict recurrence in patients diagnosed at initial stages. We identified that gains in *MAPK8* and *MKL1* genes were associated with fast progression. The *MAPK8* gene encodes the stress-activated kinase JNK1, and its activated form is associated with shorter PFS in epithelial ovarian cancer [69]. The importance of our finding is linked to the use of JNK1 inhibitors in clinical trials for other cancers [70] and to the eventual use of these inhibitors to control progression in OCCC. Interestingly, *MAPK8* gene gain was observed after progression of the Occ53 OCCC. At recurrence of Occ53, few alterations were conserved compared to the primary tumor, including amplification of *WNT7B* and *MAPK1* oncogenes (**Supplementary Fig.**S8).

## Conclusions

The low incidence of OCCC presents significant challenges to accumulate sufficient evidence to support the development of better treatments for the patients. Furthermore, the existence of geographical differences in the molecular determinants of OCCC demand collaborative efforts between research groups worldwide. Our study provides new molecular data for a geographical population barely studied in this scenery.

## Electronic supplementary material

Below is the link to the electronic supplementary material.

Supplementary Fig and Supplementary Tables

Supplementary Material 1

Supplementary Material 2

Supplementary Material 3

Supplementary Material 4

Supplementary Material 5

Supplementary Material 6

Supplementary Material 7

## Data Availability

The datasets generated and analyzed during the current study are available in the Gene Expression Omnibus (GEO) repository with accession number GSE220891.

## Abbreviations

OCCC: Ovarian clear cell carcinoma
LA: Latin America
Sxl: Simplex-like
FSl: Firestorm-like
STl: Sawtooth-like
OS: Overall survival
HRD: Homologous recombination deficiency
CN: Copy number
EOC: Epithelial ovarian cancer
HGSC: High-grade serous ovarian carcinoma
TEE: Thromboembolic event
ER: Estrogen receptor
PR: Progesterone receptor
FIGO: International Federation of Gynecology and Obstetrics
PFS: Progression-free survival
IHC: Immunohistochemistry
MMR: Mismatch repair proteins
MSI: Microsatellite instability
LRR: Log R ratio
LOH: Loss of heterozygosity
PS: “poor survival”
MB: “*MYC*-*BRCA2*”
FC: “few CN alterations”
