# Supplementary material for: Genomic landscapes of ovarian clear cell carcinoma from latin countries reveal aberrations linked to survival and progression
Source: BMC Cancer. 2023 Jul 3;23:613. doi: 10.1186/s12885-023-11095-8 (PMC10316635; doi:10.1186/s12885-023-11095-8)
Supplement: Supplementary file 1 — Supplementary Fig and Supplementary Tables [file 12885_2023_11095_MOESM8_ESM.pdf]

Supplementary Materials and Methods

Immunohistochemistry

Immunohistochemistry (IHC) was performed in tissue microarrays (TMAs) or whole sections from 10% formalin-fixed paraffin-embedded (FFPE) tissues. Antigen retrieval, primary antibodies and specific conditions are listed in **Supplementary Table S1**. The LSAB method (Dako) was performed as in conventional protocols. Nuclear staining of any intensity was considered positive expression for ARID1A, HNF1B, PTEN and mismatch repair proteins (MMR: MLH1, MSH2, MSH6, PMS2). For TP53, nuclear immunostaining was interpreted as normal (1-70% of tumor cells) or aberrant (0% or >70%). For WT1, nuclear staining in >50% of tumor cells were considered positive. For estrogen receptor (ER) and progesterone receptor (PR), positive expression was considered when >5% of tumor cells were stained.

Microsatellite Instability Analysis

Genomic DNA was extracted from OCCC FFPE tissue sections using a QIAmp DNA FFPE Tissue kit (Qiagen) and then quantified spectrophotometrically (Eppendorf Biophotometer). Microsatellite instability (MSI) was investigated using 5 mononucleotide markers (BAT-25, BAT-26, NR-21, NR-24 and NR-27). Primer sequences have been described elsewhere [1]. DNA (2-3 ng) was amplified using the Type-it Mutation Detect PCR Kit (Qiagen). Denatured PCR products were submitted to capillary electrophoresis on an Applied Biosystem 3130xL Genetic Analyzer. Fragments were analyzed using GeneMapper v.4 software (Applied Biosystems).

Detection of *PIK3CA* Gene Mutations by Real-Time PCR

The cobas® PIK3CA Mutation Test (Roche Molecular Systems Inc.) and platform were used following the manufacturer's instructions.

OncoScan Assay

The DNA extracted from the OCCC FFPE tissue sections of 26 primary tumors and one recurrent tumor was evaluated and quantified using a Denovix™ Spectrophotometer and a Quantus™ Fluorometer.

According to the manufacturer's instructions, 80 ng of genomic DNA was subjected to the SNP array OncoScan® FFPE (Thermo). The arrays were processed and loaded into the GeneChip® Scanner 3000 7G (Thermo Fisher Scientific/Affymetrix). The CEL files were generated by Affymetrix Gene Chip Command Console® software (v. 4.0).

OncoScan Data Processing

CEL files containing the raw data were processed via the Affymetrix OncoScan Console (Thermo) using the FFPE analysis workflow with NA33 (FFPPE.na33.r1) as normal reference model and applying the TuScan algorithm. Two quality control (QC) metrics provided by the software i.e., the MAPD (median of the absolute values of all pairwise differences) and the ndSNPQC (SNP QC of Normal Diploid Markers) were within bounds in 20 (76.9%) and 13 (50%) of the 26 primary-tumor samples, respectively (**Supplementary File S2**). Given that these QC markers were developed for normal diploid genomes and that heterogeneous tumor samples are expected to diverge from these, we used all available data in our analyses. The ASCAT algorithm was used to infer tumor ploidy, allele-specific copy numbers and segmentation [2]. The ASCAT package for R [3] used the following parameters: ascat.predictGermlineGenotypes(platform = "AffyOncoScan") and ascat.runAscat(gamma = 0.9). Individual LRR are presented in **Fig. 3 and Supplementary Fig. S1 and S5**. Two samples needed re-centering (Occ15 and Occ23) and four samples (Occ9, Occ23, Occ16 and Occ36) where essentially all the genome was polyploid, were re-centered to an approximate diploid version for downstream analysis (**Supplementary File S1**). The segmentation data obtained from the OncoScan console was used for the analysis in the article. CN gains/amplifications were considered when the log R ratio (LRR) was > 0.1, and CN loss/deletions were considered when the LRR was < -0.1. To define differentially aberrant segments between groups, we combined the segmented data of the samples using reduced segment algorithms of CNTools package for R. Significant differentially aberrant segments were defined using the Fisher's exact test for individual segments (P-value < 0.05). For The 4273 most variable reduced segments were used for unsupervised hierarchical clustering analysis, with Pearson distance and average-linkage. The Silhouette method was used to validate the clustering and indicated that the sample Occ36 poorly matched with the proposed cluster. The estimation of the Homology

Recombination Deficiency (HRD)-associated genomic scars (loss-of-heterozygosity: HRD-LOH, large scale transitions: HRD-LST, number of telomeric allelic imbalances: HRD-TAI and a combined score: HRD-sum) were determined on the ASCAT output using the scarHRD package for R [4]. Integrative Genome Viewer (IGV) was used to inspect the genomic profiles [5].

GISTIC

The ploidy-adjusted segmented data were corrected for purity using the tumor-purity estimation obtained from ASCAT analysis, based on the calculations performed by Zack et al. [6]. Occ36 sample was left uncorrected because tumor-purity estimation was not possible with ASCAT. Corrected data were used as the input for GISTIC2.0 (v2.0.23) [7] run in GenePattern [8] with the following parameters:confidence level =0.99; q-value = 0.25; amplification and deletion thresholds = 0.1; focal length cutoff = 0.50; arm peel: no; run broad analysis = yes). Human_Hg19.mat was used as the refgene file. The CNV file (CNV.hg19.bypos.111213.txt) was obtained from the Broad Institute.

Supplementary References

1. Buhard O, Cattaneo F, Yick FW, So FY, Friedman E, Flejou JF, et al. Multipopulation analysis of polymorphisms in five mononucleotide repeats used to determine the microsatellite instability status of human tumors. Journal of Clinical Oncology. 2006;24:241–51.

2. Van Loo P, Nordgard SH, Lingjærde OC, Russnes HG, Rye IH, Sun W, et al. Allele-specific copy number analysis of tumors. Proc Natl Acad Sci U S A. 2010;107:16910–5.

3. Ross EM, Haase K, Van Loo P, Markowetz F. Allele-specific multi-sample copy number segmentation in ASCAT. Bioinformatics. 2021;37:1909–11.

4. Sztupinszki Z, Diossy M, Krzystanek M, Reiniger L, Csabai I, Favero F, et al. Migrating the SNP array-based homologous recombination deficiency measures to next generation sequencing data of breast cancer. NPJ Breast Cancer. 2018;4:8–11.

5. Thorvaldsdóttir H, Robinson JT, Mesirov JP. Integrative Genomics Viewer (IGV): High-performance genomics data visualization and exploration. Brief Bioinform. 2013;14:178–92.

6. Zack TI, Schumacher SE, Carter SL, Cherniack AD, Saksena G, Tabak B, et al. Pan-cancer patterns of somatic copy number alteration. Nature Genetics 2013 45:10. 2013;45:1134–40.

7. Mermel CH, Schumacher SE, Hill B, Meyerson ML, Beroukhim R, Getz G. GISTIC2.0 facilitates sensitive and confident localization of the targets of focal somatic copy-number alteration in human cancers. Genome Biol. 2011;12:1–14.

8. Reich M, Liefeld T, Gould J, Lerner J, Tamayo P, Mesirov JP. GenePattern 2.0. Nat Genet. 2006;38:500–1.

Supplementary Tables

Supplementary Table S1 Antibodies and conditions for IHC reactions

Antibody	Supplier	Catalog #	Dilution	Antigen retrieval	Incubation (min)
HNF1B	Sigma		pure 1/100	low pH, 97°C, 30 min	30
WT1	DAKO	Clone GF-H2	prediluted	high pH, 97°C, 30 min	18
ER	DAKO	Clone FP1	prediluted	high pH, 97°C, 30 min	12.5
PR	DAKO	Clone PgR 1294	prediluted	high pH, 97°C, 30 min	25
ARID1A-L	Abcam	EPR13501-73	pure 1/100	low pH, 97°C, 30 min	20
TP53	DAKO	Clone DO-7	prediluted	high pH, 97°C, 30 min	25
PTEN	DAKO	Clone 6H2.1	pure 1/100	high pH, 97°C, 30 min	20
MLH1	DAKO	Clone ES05	prediluted	high pH, 97°C, 30 min	35
MSH2	DAKO	Clone FE11	prediluted	high pH, 97°C, 30 min	20
MSH6	DAKO	Clone EP49	prediluted	high pH, 97°C, 30 min	20
PMS2	DAKO	Clone EP51	prediluted	high pH, 97°C, 30 min	40

Supplementary Table S2 Univariate analysis of OCCC

Parameter	n	HR	95% CI	*P*-value
**OS**				
Figo stage				
advanced (III/IV)	21	5.803	2.608-12.91	1.64e-05 ***
initial (I/II)	39	1		
platinum				
resistant	35	21.09	7.338-60.61	1.51e-08 ***
sensitive	18	1		
endometriosis				
yes	16	0.5675	0.2155-1.494	0.252
no	44	1		
TEEs				
yes	13	2.932	1.328-6.475	0.00777 **
no	47	1		
**PFS**				
Figo stage				
advanced (III/IV)	21	6.816	3.3-14.08	2.16e-07 ***
initial (I/II)	39	1		
endometriosis				
yes	16	0.422	0.1746-1.02	0.0554
no	44	1		
TEEs				
yes	13	2.044	0.9983-4.183	0.0505
no	47	1		

OS Overall survival, *PFS* Progression-free survival, *FIGO* International Federation of Gynecology and Obstetrics, *TEE* thromboembolic event

Supplementary Table S3 Relationship between *PIK3CA* mutation status in primary tumor samples and OCCC features

Parameter	*PIK3CA* wild-type	*PIK3CA* mutant	*P*-value
FIGO stage, n			
Initial (I/II)	17	12	0.0487 *
Advanced (III/IV)	15	2	
ARID1A expression, n			
negative	5	8	0.0114 *
positive	22	5	

FIGO International Federation of Gynecology and Obstetrics

Supplementary Figures

Supplementary Fig. S1 Individual genomic profiles of OCCC.

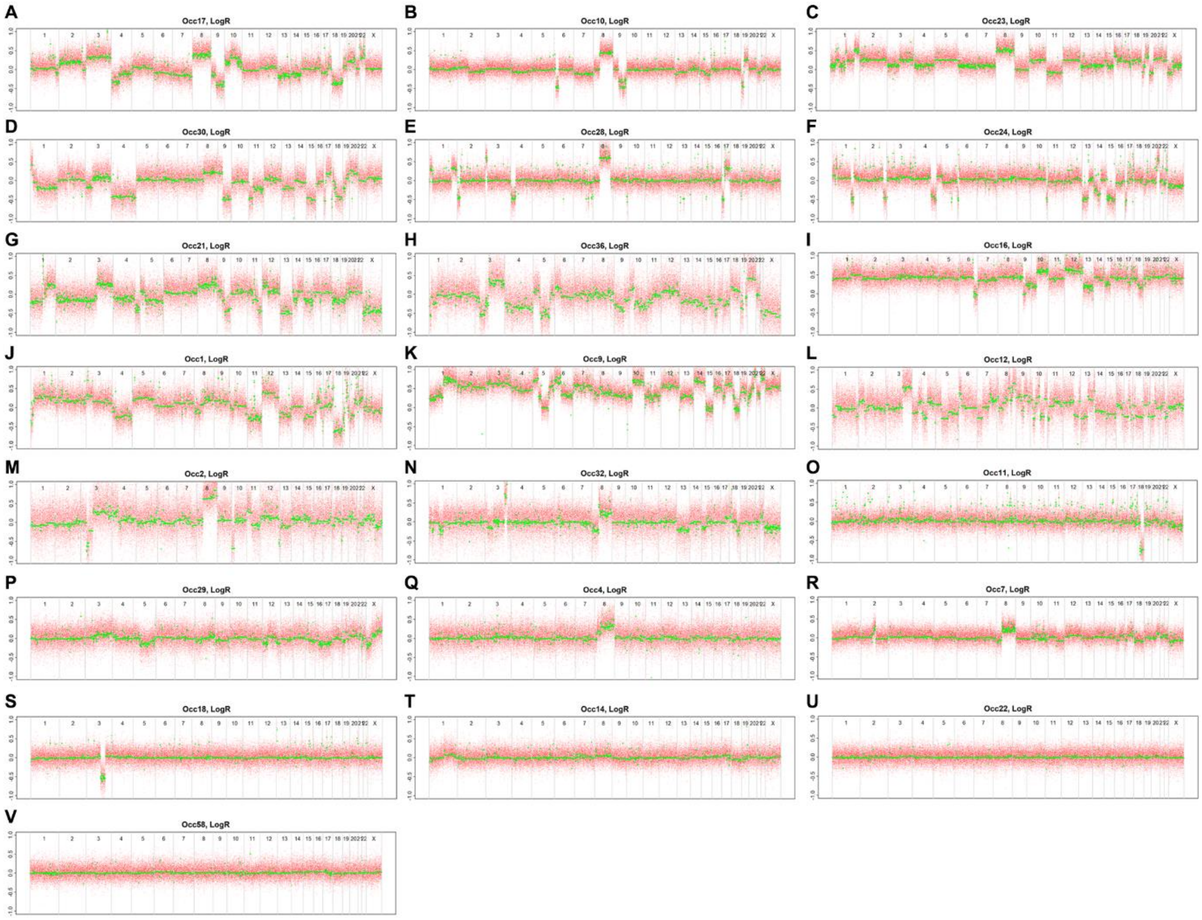

Supplementary Fig. S1 Individual genomic profiles of OCCC. **A-V** Segmentation data of primary-tumor OCCC samples were used for the ASCAT algorithm. The graphs show individual log2 ratio profiles. Additional profiles are shown in Fig. 1 and Supplementary Fig. S8.

Supplementary Fig. S2 OCCC groups based on HRD-sum levels were not related to OS or PFS.

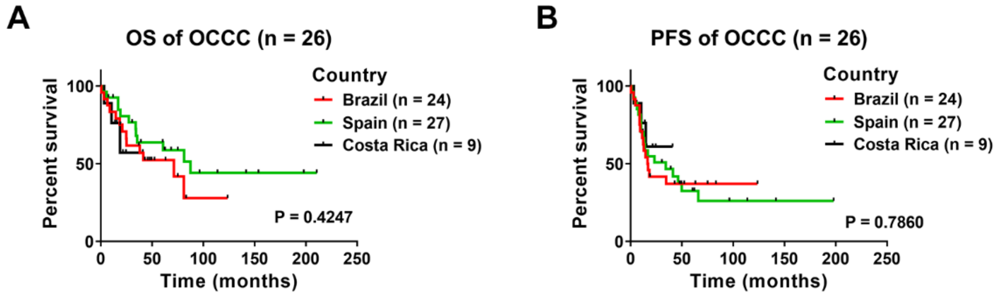

Supplementary Fig. S2 OCCC groups based on HRD-sum levels were not related to OS or PFS. **A** OS analysis based on the country of origin of the OCCC cases. **B** PFS analysis based on the country of origin of the OCCC cases.

Supplementary Fig. S3 Multivariate analysis for OCCC.

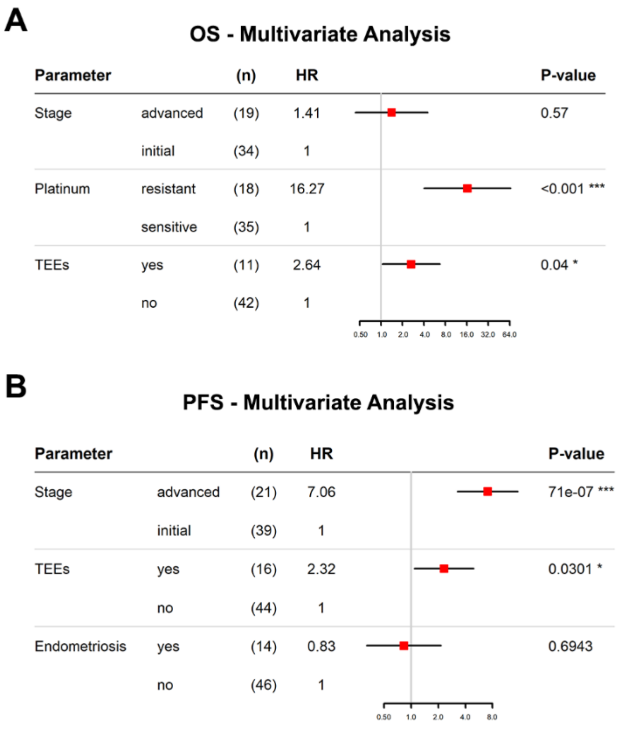

Supplementary Fig. S3 Multivariate analysis for OCCC. Multivariate analysis for (**A**) OS and (**B**) PFS using parameters with a *P*-value < 0.06 in univariate analysis (**Supplementary Table S2**) as covariates.

Supplementary Fig. S4 Copy number alterations in OCCC.

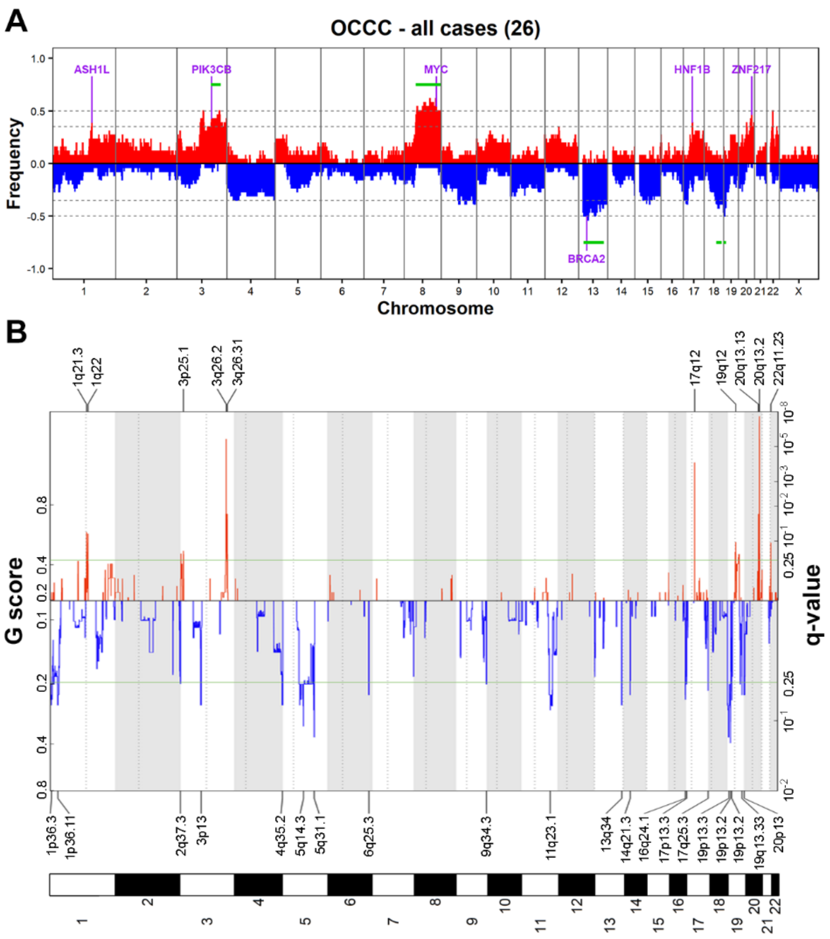

Supplementary Fig. S4 Copy number alterations in OCCC. **A** Frequency of CN alterations in 26 OCCC primary tumors analyzed by OncoScan. Recurrently altered genes and regions (green lines) are indicated. **B** GISTIC analysis of 26 OCCC primary tumors. Significantly altered peaks of amplification and deletion (G score < 0.25) are indicated.

Supplementary Fig. S5 OCCC clusters based on genomic patterns are not related to OS or PFS.

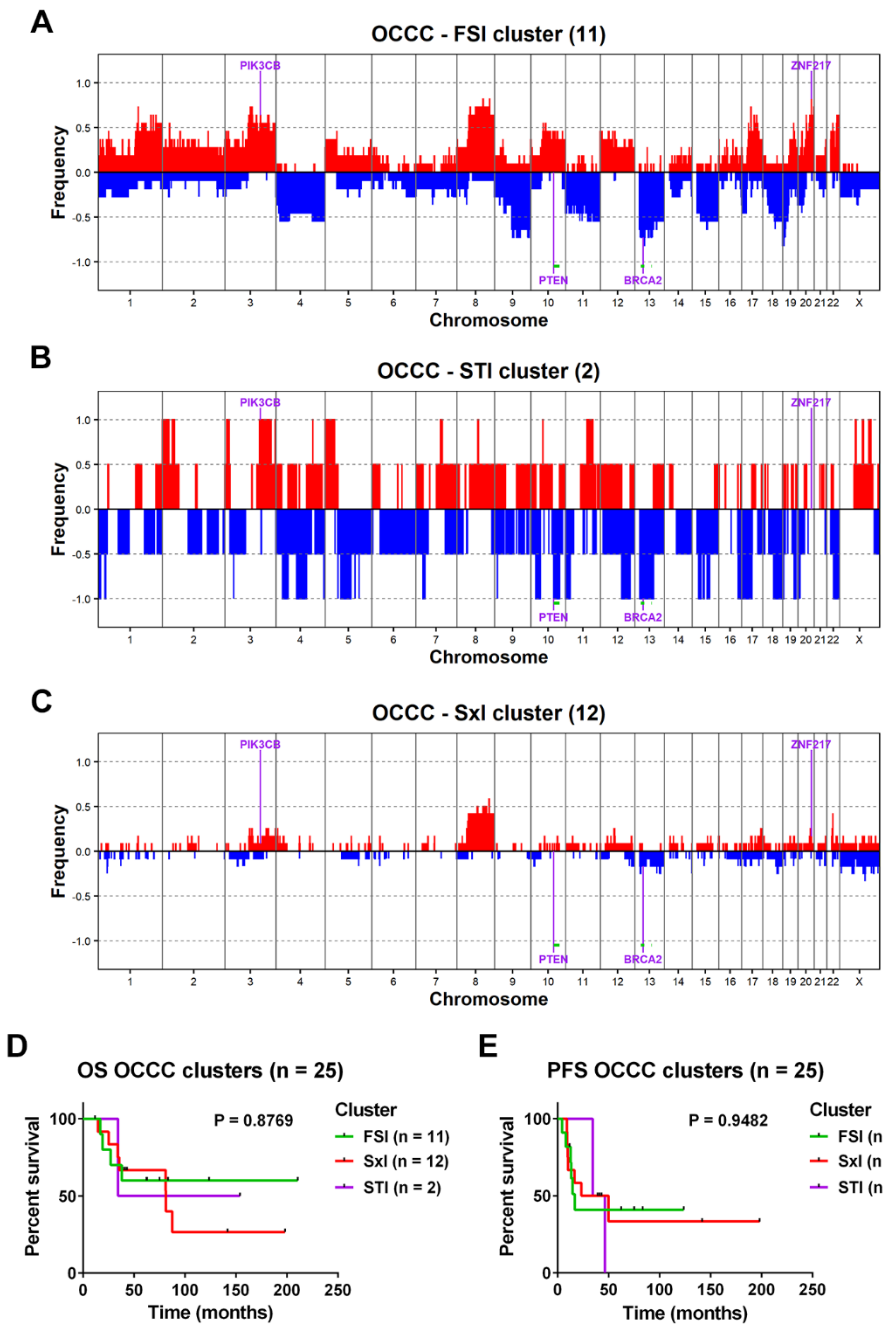

Supplementary Fig. S5 OCCC clusters based on genomic patterns are not related to OS or PFS. **A-C** Frequency plots were generated for the (**A**) FSl, (**B**) STl and (**C**) Sxl clusters. Significantly altered genes and regions (green line) are indicated. **D** OS analysis for OCCC clusters. **E** PFS analysis of OCCC clusters.

Supplementary Fig. S6 OCCC groups based on HRD-sum levels were not related to OS or PFS.

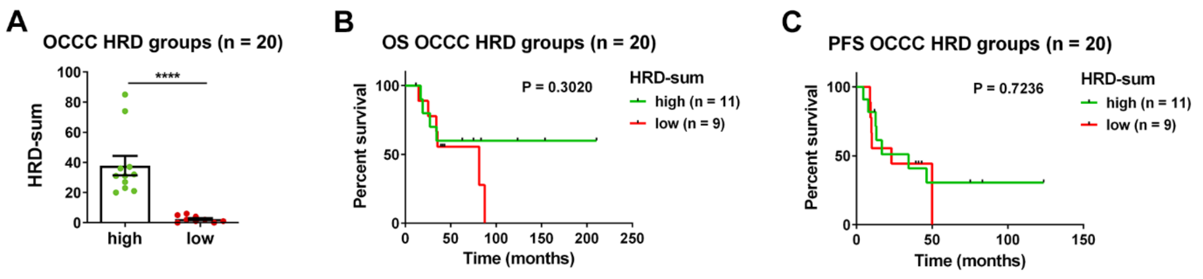

Supplementary Fig. S6 OCCC groups based on HRD-sum levels were not related to OS or PFS. A HRD-sum levels for each group. ****P-value < 0.0001 for Mann-Whitney test. **B** OS analysis for groups of OCCC with different HRD-sum levels. **C** PFS analysis for groups of OCCC with different HRD-sum levels.

Supplementary Fig. S7 Characteristics of OS groups.

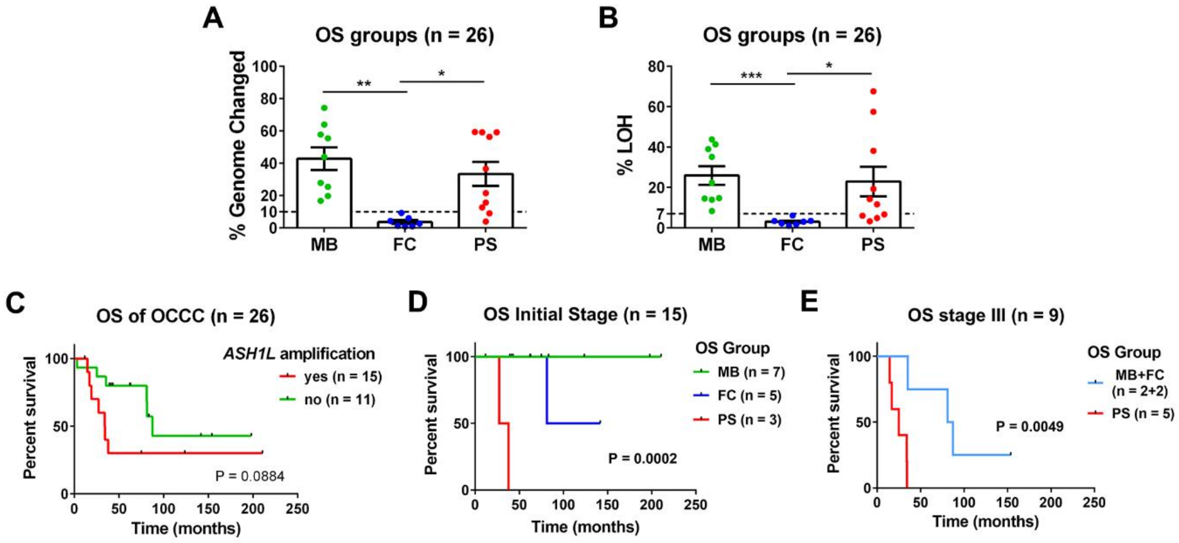

Supplementary Fig. S7 Characteristics of OS groups. **A** Graph showing the percentage of genome changes in each OS group. **B** Graph showing the percentage of LOH regions within the genome of each OS group. Significant differences were analyzed by the Kruskal-Wallis test. **C** Survival analysis for groups showing, or not, *ASH1L* gene amplification. **D** Survival analysis for initial-stage OCCCs based on OS groups. **E** Survival analysis for stage III OCCCs based on OS groups. Since few stage III samples were not PS samples, they were combined into a non-PS group (MB+FC).

Supplementary Fig. S8 Genomic alterations in one recurrent OCCC sample.

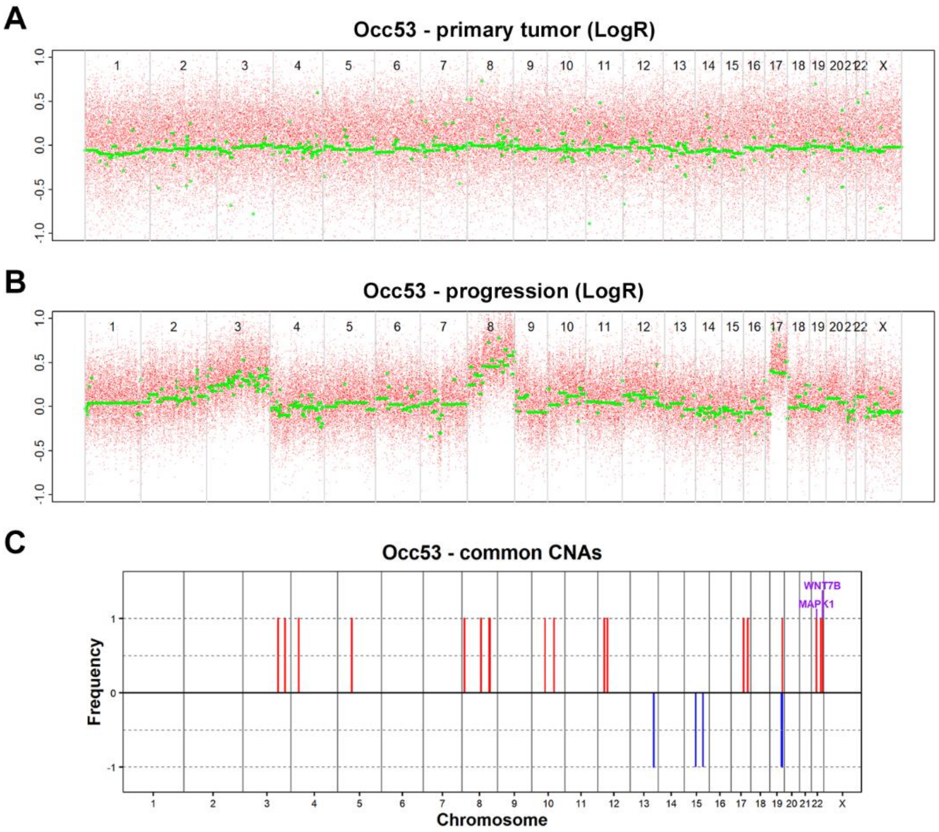

Supplementary Fig. S8 Genomic alterations in one recurrent OCCC sample. **A, B** Log2 ratio profiles obtained with ASCAT for a (**A**) primary-tumor OCCC sample and its (**B**) recurrence. **C** Plot showing the common CN alterations and genes for the two samples.

Additional Files

Additional file 1: Supplementary File S1. Clinical and molecular data for the 60 OCCC samples (Excel file). Sheet1 shows the clinical data for the whole cohort. Sheet2 shows the molecular data obtained from the primary-tumor OCCC samples. Sheet3 shows the molecular data obtained from the samples after recurrence. Occ53 has paired primary and recurrence samples.

Additional file 2: Supplementary File S2. Quality control and recentering of OncoScan samples (Excel file). The quality parameters MAPD and ndSNPQC for OCCC primary tumor OncoScan analysis were obtained with the OncoScan console (Affymetrix/Thermo). Samples that did not pass the recommended QC threshold are indicated. Recentering was necessary for two samples, and a chromosome that was mostly diploid was used as a reference. We adapted OncoScan data of polyploid samples to a diploid version (analogous to aCGH data) by manual recentering, as indicated. Sheet2 shows the QC parameters for the recurrence of Occ53.

Additional file 3: Supplementary File S3. Summary of the OncoScan results for 26 primary-tumor OCCC samples (Excel file). Sheet2 shows the corresponding data for the recurrence of Occ53.

Additional file 4: Supplementary File S4. Frequency of copy number alterations in OCCC (Excel file). CNVs of 26 OCCC primary tumors were analyzed by OncoScan. Sheet1 shows the chromosomal regions with gains in at least 9 samples (35%). Sheet2 shows the chromosomal regions with loss in at least 9 samples (35%). Regions were defined as contiguous segments with at least 9 samples showing copy number alterations in the same direction. Since segments within a region could be altered in different numbers of cases, we represented the frequency and number of cases as a weighted average (*). Sheet3 shows the frequency of alterations at the gene level. Sheet4 shows the frequency of alterations of individual segments. CN alterations that are registered in the Database of Genomic Variants (http://dgv.tcag.ca/dgv/app/home) are indicated.

Additional file 5: Supplementary File S5. Results of GISTIC analysis of OCCC (Excel file). Segmented data obtained by OncoScan from 26 OCCC primary tumors were used to identify amplification and deletion peaks. A q-value < 0.25 was used to define significant peaks.

Additional file 6: Supplementary File S6. Differentially aberrant segments in the OS groups (Excel file). CN aberrations from the OS group (MB, FC and PS) were compared. Comparisons were performed for individual groups or for a group vs. the combination of the other two groups. Segments with *P*-values < 0.05 in Fisher's exact test were selected. Regions were defined as contiguous significant segments, and the weighted average of the values of the segments were calculated. Sheet1 contains the regions of significant gains. Sheet2 contains regions of significant losses. Sheet3 contains individual genes showing significant gains. Sheet4 contains individual genes showing significant losses.

Additional file 7: Supplementary File S7. Differentially aberrant segments in samples that recurred or not (Excel file). CN aberrations were compared, and segments with *P*-values < 0.05 in Fisher's exact test were selected. Regions were defined as contiguous significant segments, and the weighted average of the values of the segments were calculated. Sheet1 contains the regions of significant gains. Sheet2 contains regions of significant losses. Sheet3 contains individual genes showing significant gains. No individual gene showed significant losses.
